# Supplementary material for: Signature of quantum criticality in cuprates by charge density fluctuations
Source: Nat Commun. 2023 Nov 8;14:7198. doi: 10.1038/s41467-023-42961-5 (PMC10632404; doi:10.1038/s41467-023-42961-5)
Supplement: Supplementary file 1 — Supplementary Information [file 41467_2023_42961_MOESM1_ESM.pdf]

Supplementary Information for

Signature of quantum criticality in cuprates by charge density fluctuations

Riccardo Arpaia[1,*], Leonardo Martinelli[2], Marco Moretti Sala[2], Sergio Caprara[3,4], Abhishek Nag[5], Nicholas B. Brookes[6], Pietro Camisa[2], Qizhi Li[7], Qiang Gao[8], Xingjiang Zhou[8], Mirian Garcia-Fernandez[5], Ke-Jin Zhou[5], Enrico Schierle[9], Thilo Bauch[1], Ying Ying Peng[7], Carlo Di Castro[3], Marco Grilli[3,4], Floriana Lombardi[1], Lucio Braicovich[2,6], Giacomo Ghiringhelli[2,10,*]

[1] *Quantum Device Physics Laboratory, Department of Microtechnology and Nanoscience, Chalmers University of Technology, SE-41296 Göteborg, Sweden*

[2] *Dipartimento di Fisica, Politecnico di Milano, Piazza Leonardo da Vinci 32, I-20133 Milano, Italy*

[3] *Dipartimento di Fisica, Università di Roma "La Sapienza" P.^{le} Aldo Moro 5, I-00185 Roma, Italy*

[4] *CNR-ISC, via dei Taurini 19, I-00185 Roma, Italy*

[5] *Diamond Light Source, Harwell Campus, Didcot OX11 0DE, United Kingdom*

[6] *ESRF, The European Synchrotron, 71 Avenue des Martyrs, F-38000 Grenoble, France*

[7] *International Center for Quantum Materials, School of Physics, Peking University, CN-100871 Beijing, China*

[8] *Beijing National Laboratory for Condensed Matter Physics, Institute of Physics, Chinese Academy of Sciences, CN-100190 Beijing, China*

[9] *Helmholtz-Zentrum Berlin für Materialien und Energie, Albert-Einstein-Straße 15, D-12489 Berlin, Germany*

[10] *CNR-SPIN, Dipartimento di Fisica, Politecnico di Milano, Piazza Leonardo da Vinci 32, I-20133 Milano, Italy*

* Correspondence to: riccardo.arpaia@chalmers.se; giacomo.ghiringhelli@polimi.it

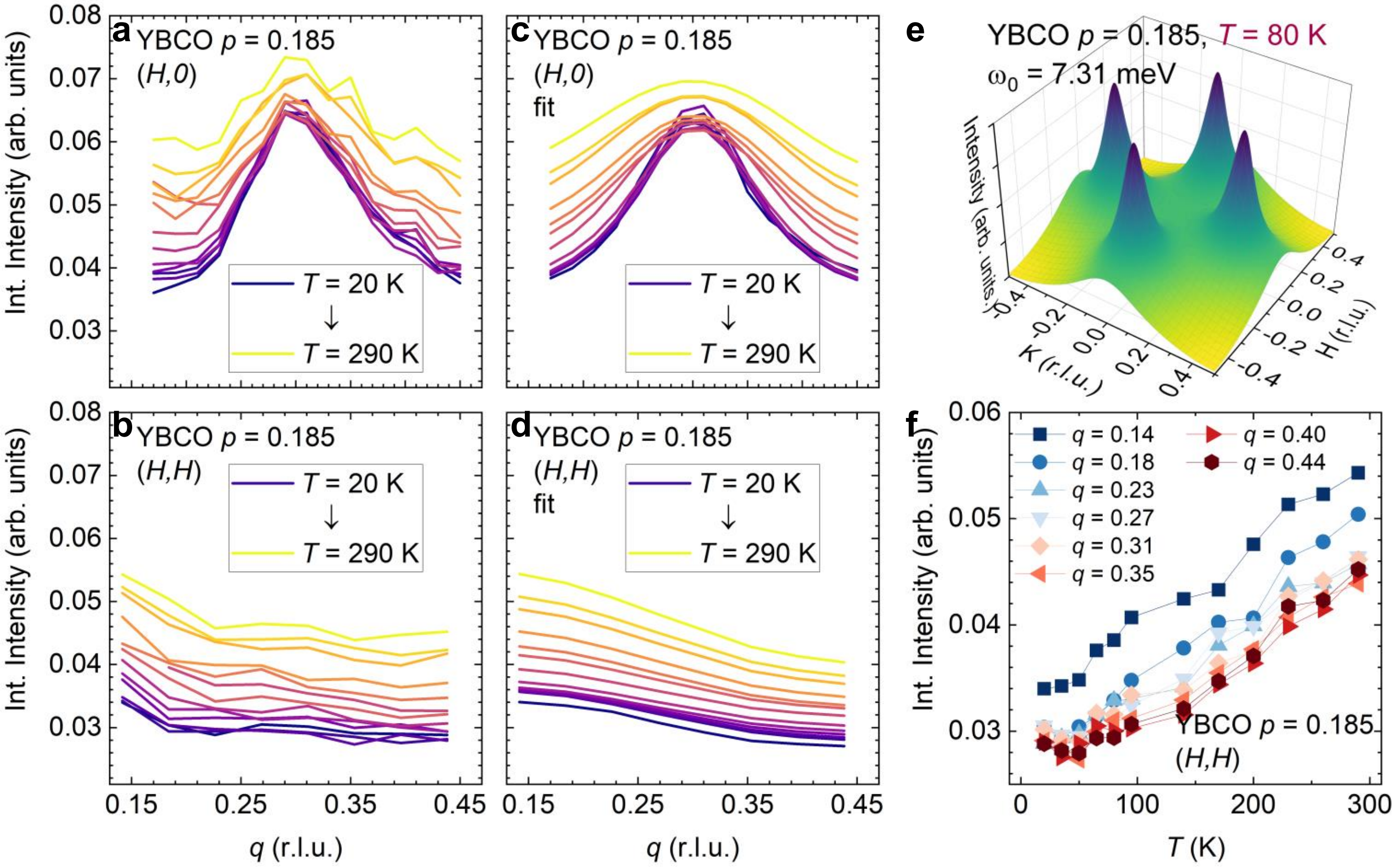

Supplementary Fig.1: **Medium resolution RIXS measurements on YBCO (*p*=0.185). a,b** Integrated quasi elastic intensity of the spectra measured respectively along the (**a**) (*H*,*0*) and (**b**) (*H*,*H*) directions is shown as a function of the momentum *q* at several temperatures *T*. The contour plot of these scans is in Fig. 2b,c. **c,d,** Calculated scans, determined via a global fit of the curves presented in panels (a) and (b). The contour plot of these scans is in Fig. 2d,e. **e,** Schematics of the four equivalent peaks in the Brillouin zone used to perform the global fit. Here, the used parameters are to fit the (*H*,0) and (*H*,*H*) scans at 80 K. It appears evident that the peaks centered along the (*H*,0) and (0,*K*) directions are so broad as to influence the intensity along the diagonal. **f,** The quasi elastic intensity is plotted here as a function of the temperature for different momenta *q* along the (*H*,*H*) direction. At every *q* the intensity rises with a similar slope. The vertical shift at small *q* is a consequence of the specular peak centered at Γ.

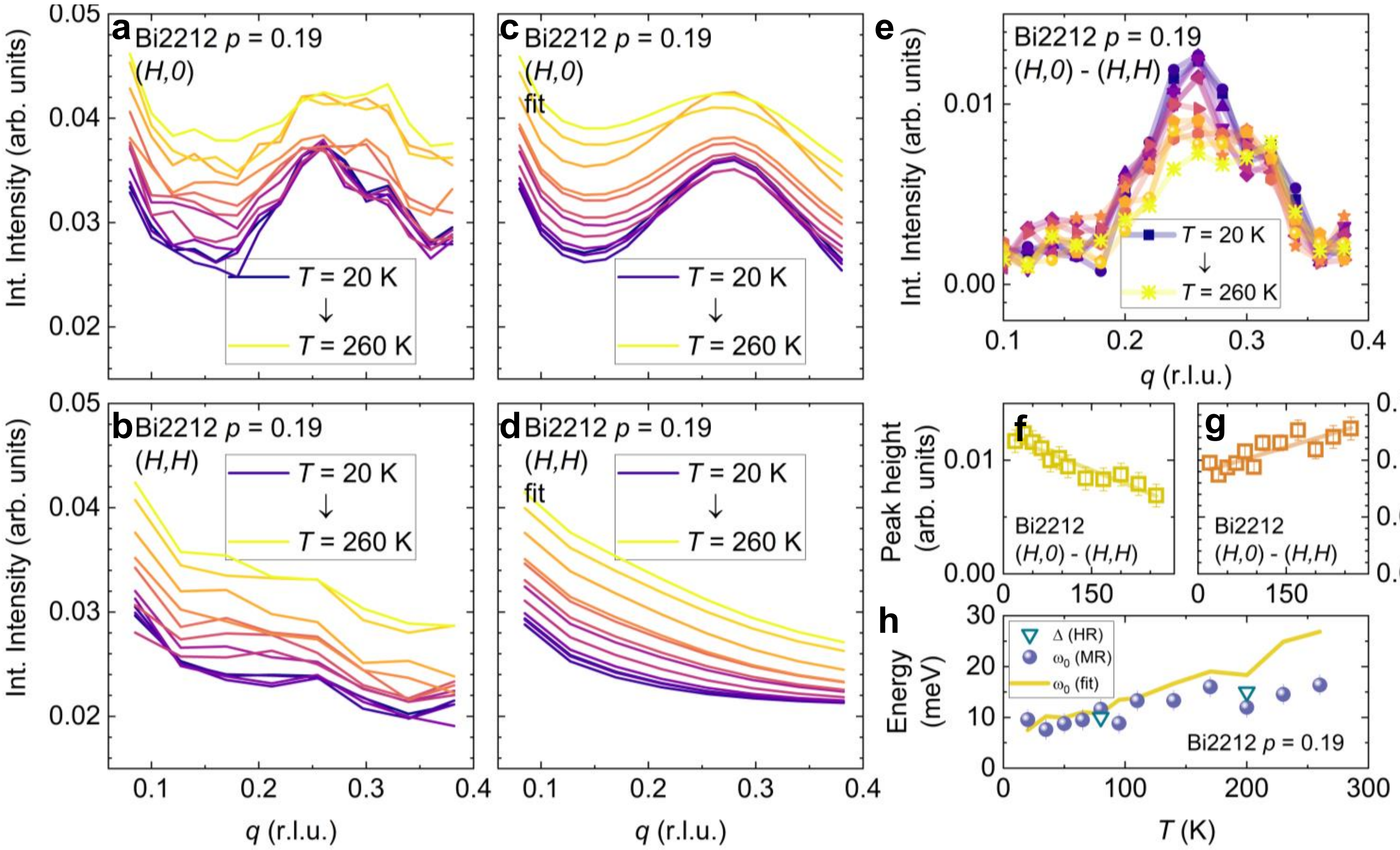

Supplementary Fig. 2: **Medium resolution RIXS measurements on Bi2212 (*p*=0.19). a,b** Integrated quasi elastic intensity of the spectra measured respectively along the (**a**) (*H*,*0*) and (**b**) (*H*,*H*) directions is shown as a function of the momentum *q* at several temperatures *T*. **c,d,** Calculated scans, determined via a global fit of the curves presented in panels (a) and (b). The experimental data are fitted considering ω_0 varying with temperature in the range 7-25 meV, $\overline{\omega}$ = 64 meV, ν_0 = 1.52 eV(r.l.u.)$^{-2}$, $\gamma \approx 1$ around T_c. **e,** CDF peaks at several temperatures, determined as the difference between the (*H*,*0*) and the (*H*,*H*) scans. Each peak has been fitted using a single Lorentzian, to get more insight on the *T*-dependence of CDF. **f,g,** The height and FWHM of the single Lorentzian profiles used to fit the data in panel (**e**) are plotted vs temperature. The error bars represent the 95% confidence interval of the Lorentzian fit. The solid line is a linear fit of the data. **h,** The energy Δ, extracted from the HR spectra, and the frequency ω_0, determined from the medium resolution spectra by the FWHM of the CDF profiles, are plotted as a function of the temperature respectively as triangles and circles. The frequency ω_0, determined by the global fit (solid line), is in fairly good qualitative agreement with the experiment, showing an increase as *T* raises.

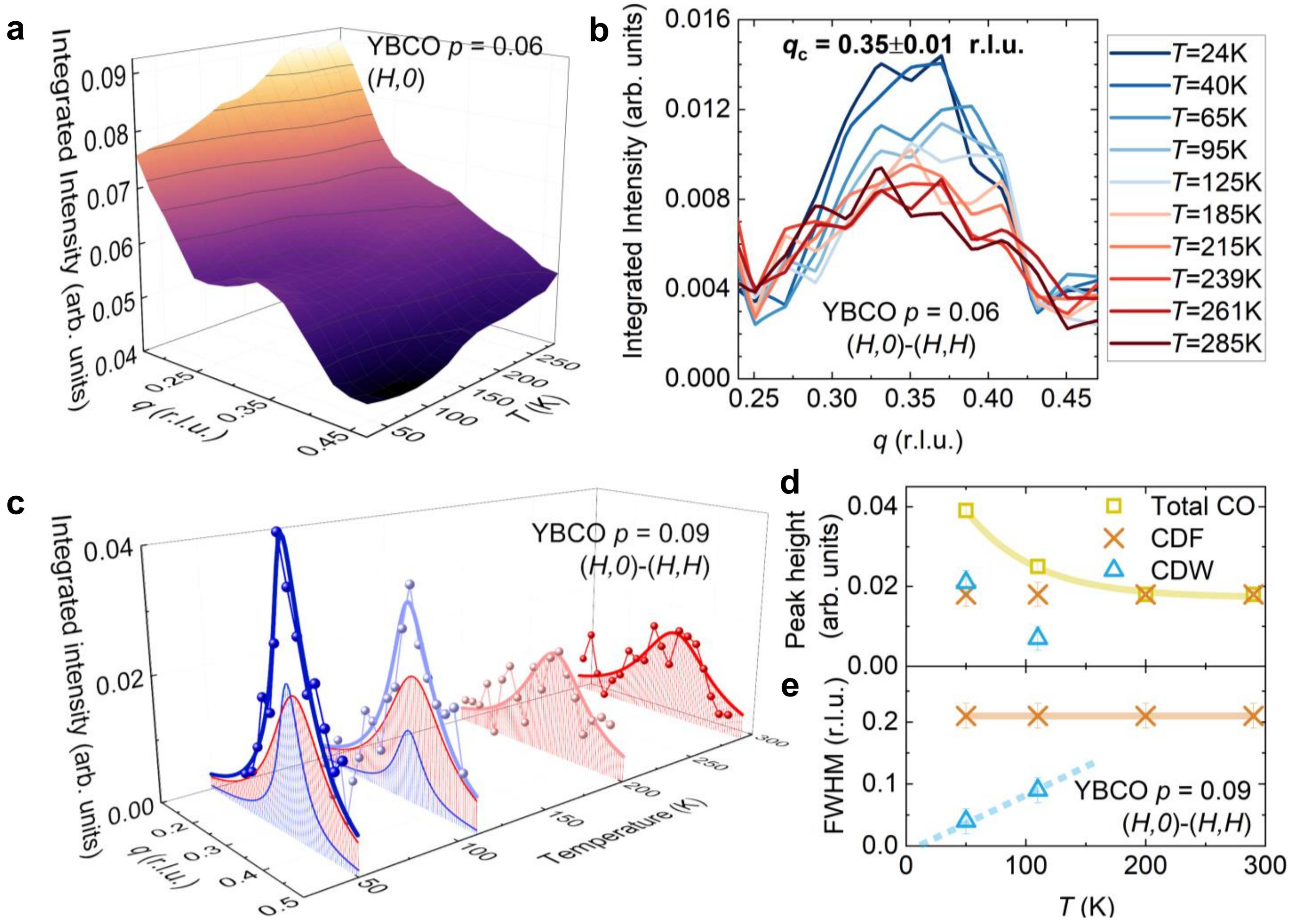

Supplementary Fig. 3: **Medium resolution RIXS measurements on two underdoped YBCO samples.** **a,** 3D-surface of the q scans measured along the (H,0) directions at different temperatures on the YBCO (p=0.06). Following the T axis, one can notice the increase of the quasi-elastic intensity as the temperature increases. **b,** CDF peak, i.e. difference (H,0)-(H,H), of the YBCO (p=0.06), at several temperatures. **c,** CDF peak of the YBCO (p=0.09) at several temperatures. Here, we have only measured at four temperatures. Below 110 K, a rather intense, strongly T-dependent, CDW signal (blue Lorentzians) appears, in addition to the mildly T-dependent, broad CDF peak (red Lorentzians) already present at room temperature. **d,e,** Temperature evolution of the CDF height (**d**) and width (**e**). The presence of the CDW makes very difficult to single out and determine the T-evolution of the CDF peak. For simplicity, we have supposed the latter (crosses) T-independent, in agreement with the procedure already followed in ref. 1. About the CDW peak (triangles), as the temperature decreases and T_c is approached, its intensity grows and its FWHM becomes narrower. In particular, the linear extrapolation to zero of the CDF width provides an estimate of a finite temperature, below which, in the absence of superconductivity, ξ would diverge. This behavior, typical of critical phenomena, characterized by the divergence of a spatial correlation length, is very different from what observed for the CDF (see Fig. 2h).

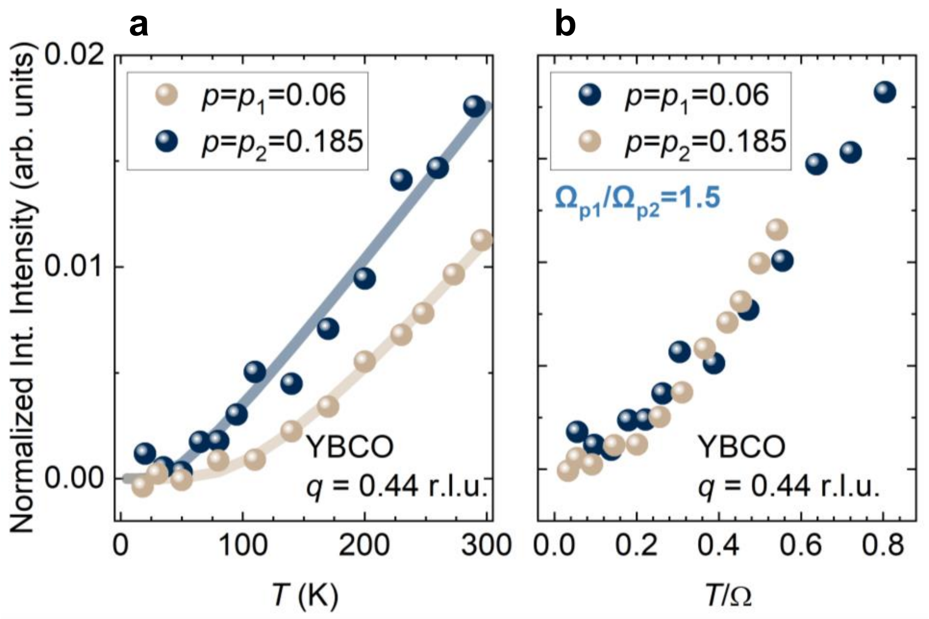

Supplementary Fig. 4: **Robustness of the doping dependence of the CDF energy along (*H*,*H*) via the Bose fit. a,** The quasi-elastic intensity measured at q=0.44 r.l.u. along the (H,H) direction is plotted as a function of the temperature for the strongly underdoped (p=0.06, grey dots) and for the slightly overdoped (p=0.185, blue dots) YBCO samples. To generalize the fit procedure presented in Fig. 4a,b, and show that the results are independent of the additive and multiplicative constants present in the Bose function we used, here we have presented the data after subtracting for both samples the zero-temperature contribution $A+I_0$, deriving from CDF and from the specular peak. It appears evident that the temperature dependence of the quasi-elastic intensity is doping dependent, being the signal rise much steeper at higher doping. **b,** The two dataset of panel (**a**) are plotted in terms of the reduced temperature T/Ω. Using this variable, the Bose function takes its universal form, and all the different curves should collapse with an appropriated choice of Ω. In our case, the two curves collapse on each other if the ratio between the energy Ω at p=0.06 and that at p=0.185 is 1.5. This ratio corresponds to that we have found in the main text, using the Bose function including the A and the I_0 constants, and it is very close to the ratio we have found for the energy Δ at $H=H_{CDF}$ from the high resolution spectra.

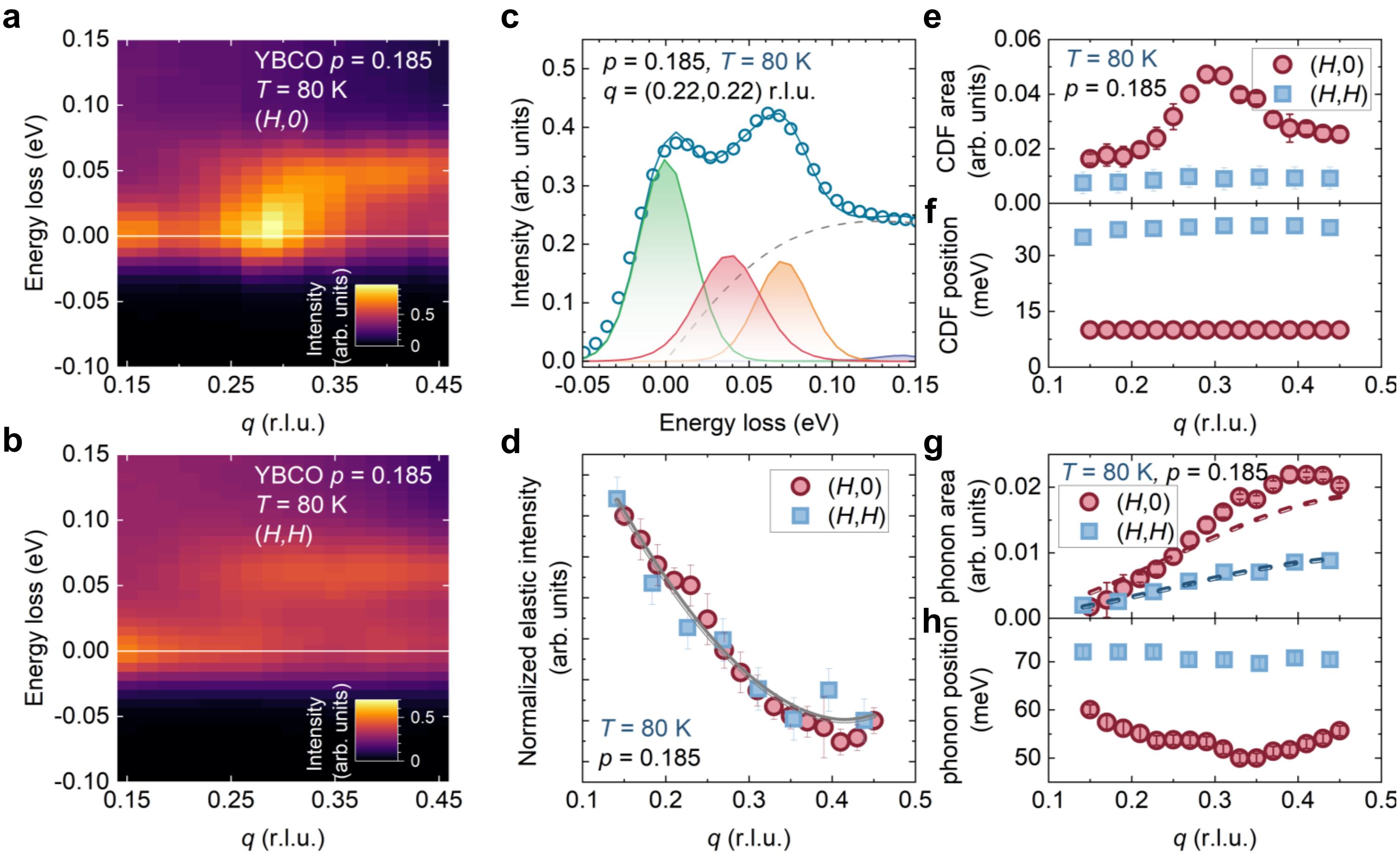

Supplementary Fig. 5: **High resolution RIXS measurements on YBCO (*p*=0.185) and comparison between the (*H*,0) and the (*H*,*H*) directions. a,b,** Intensity maps of the low-energy region of the RIXS spectra measured at several momenta along both the (*H*,0) (**a**) and the (*H*,*H*) (**b**) directions. The measurements are all taken at T=80 K. **c,** Fit of a RIXS spectrum at a representative momentum along the (*H*,*H*) direction. The green, red, orange, blue Gaussians and the region below the grey dashed line represent respectively the pure elastic, the CDF, the bond-stretching phonon modes, the phonon overtones and the paramagnons. We have therefore calculated as a function of q the position and intensity of all these Gaussians, finding strong differences between the (*H*,0) and the (*H*,*H*) directions, as shown in the next panels. **d,** Area of the elastic line along the (*H*,0) (circles) and the (*H*,*H*) (squares) directions: the q dependences are very similar. **e,** Area of the CDF peak along the (*H*,0) (circles) and the (*H*,*H*) (squares) directions: a q-resonance at q_c is present only along the (*H*,0) direction, while along the (*H*,*H*) direction only a featureless background is present. **f,** Position of the CDF peak along the (*H*,0) (circles) and the (*H*,*H*) (squares) directions: the energy along the (*H*,*H*) direction is higher than that along the (*H*,0) direction, being around 35 meV at any q. Along the (*H*,0) direction the energy is instead 10 meV, as determined at q close to q_c. We have fixed this value also at q far from q_c, to prevent instabilities in the fit. **g,** Area of the bond-stretching phonon peak along the (*H*,0) (circles) and the (*H*,*H*) (squares) directions: along the (*H*,*H*) direction the q-dependence is, as expected[2,3], proportional to $\sin^2 \pi q$; viceversa, along the (*H*,0) direction an intensity anomaly occurs, consequently to the observed softening[4,5,6,7]. **h,** Bond-stretching phonon dispersion along the (*H*,0) (circles) and the (*H*,*H*) (squares) directions: the softening is rather pronounced, with a maximum around q = 0.35 r.l.u., along the (*H*,0) direction, while it is negligible (< 2 meV) along the (*H*,*H*) direction.

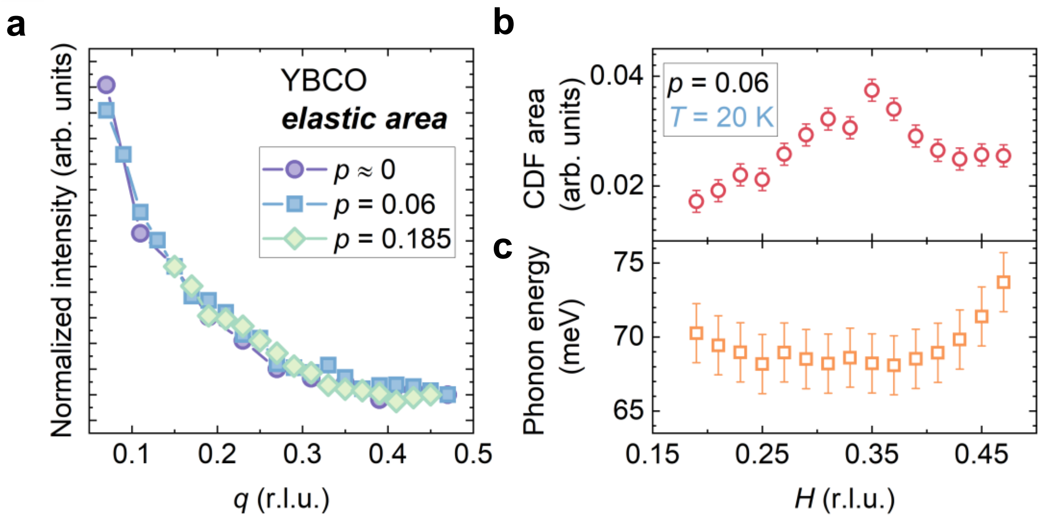

Supplementary Fig. 6: **High resolution RIXS measurements on strongly underdoped YBCO samples, (*H*,0) direction. a,** Area of the pure elastic Gaussian vs momentum for an undoped ($p\approx0$, circles), a strongly underdoped (p=0.06, squares) and a slightly overdoped (p=0.185, diamonds) YBCO. The q dependences are very similar in all the samples, which confirm the goodness of the fit presented in Fig.1 and Supplementary Fig. 5 in singling out this contribution from the higher energy, CDF contribution. **b,** Area of the CDF peak extracted from the fit on the high resolution spectra measured on the strongly underdoped YBCO. The peak is centered at q_c = 0.35 r.l.u., in agreement with the result on the medium resolution RIXS spectra (see Supplementary Fig. 3). **c,** Bond-stretching phonon dispersion determined from the high resolution RIXS spectra on strongly underdoped YBCO. A softening occurs, although much milder than in the slightly overdoped sample (see Supplementary Fig. 5h).

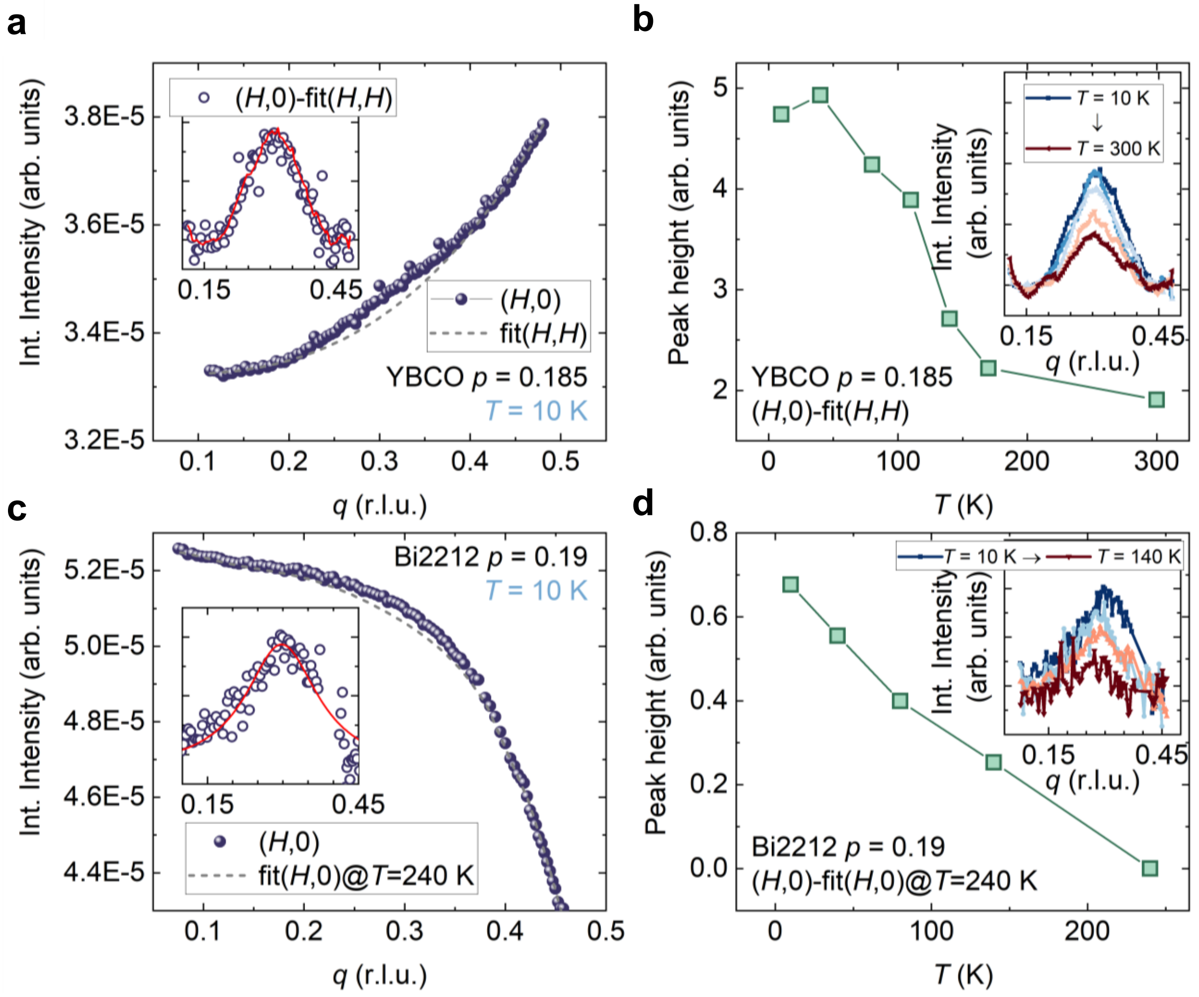

Supplementary Fig. 7: **EI-RXS measurements on slightly overdoped YBCO and Bi2212. a,** To extract the CDF peak, we have used a procedure analogous to that followed for medium resolution RIXS measurements. First, we have measured the (*H*,0) scan (blue spheres) on the slightly overdoped YBCO. After that, we have measured the (*H*,*H*) scan as a background. Here, the comparison between (*H*,0) and (*H*,*H*) scans is less immediate than for RIXS. EI-RXS integrates indeed on an energy loss range much wider than the quasi-elastic one used for RIXS, which contains magnetic, charge transfer and inter-orbital excitations. Consequently, the (*H*,*H*) scan presents a slope and absolute values rather different from those along the (*H*,0) direction. However, it can be fitted using a polynomial of degree 3, which also fits the extremes of the (*H*,0) scan, far from q_{CDF} (see dashed line). In the inset, the CDF contribution is obtained therefore by the difference between the (*H*,0) scan and the polynomial function which fits the (*H*,*H*) scan. The resulting peak has q_{CDF} and FWHM in agreement with that determined with RIXS (see Supplementary Fig. 1a). **b,** The height of the CDF peaks is plotted versus temperature. The CDF peak, shown in the inset, presents a mild *T*-dependence, becoming wider (as a consequence of the energy rise) when *T* increases. **c,d** Same as panels (**a**) and (b), but for slightly overdoped Bi2212. Here, the only difference is that to isolate the CDF contribution we have not used the (*H*,*H*) scan as background. Viceversa, we have used the fit of the (*H*,0) scan measured at the highest temperature (240 K). This is a procedure commonly used for years to study the *T*-dependence of CDW, even though it intrinsically removes the high temperature component of charge order.

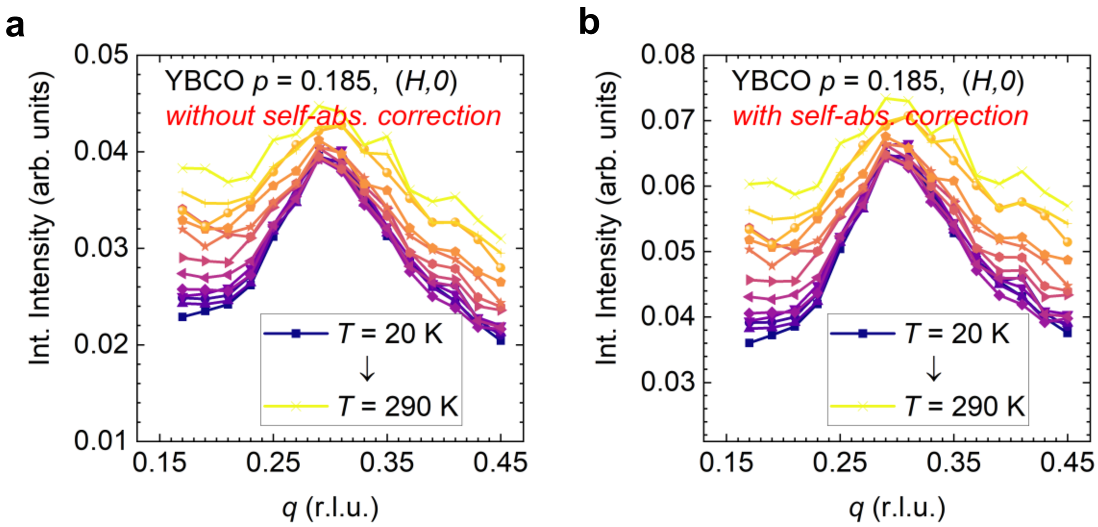

Supplementary Fig. 8: **Effect of the self-absorption corrections on the quasi-elastic RIXS intensity.** The integrated quasi elastic intensity of the medium energy resolution RIXS spectra measured along the (*H*,*0*) direction of the YBCO (p=0.185) sample is shown as a function of the temperature **a,** before and **b,** after the self-absorption correction. It is evident that the FWHM, as well as the wave vector q_{CDF}, of the CDF peaks are unchanged.

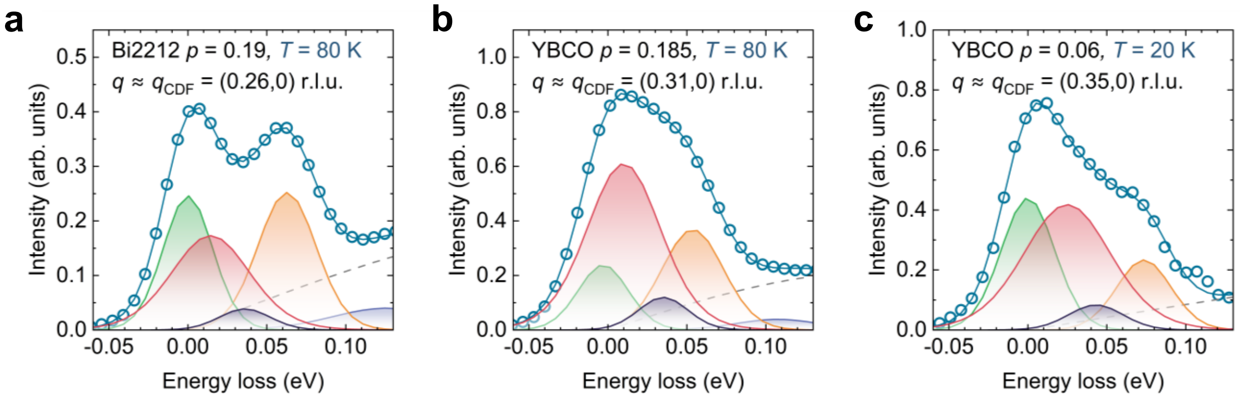

Supplementary Fig. 9: **Multi-peak fit of the high-resolution spectra considering the buckling phonons.** Fit of a high-resolution RIXS spectrum at $q \approx q_{\mathrm{CDF}}$ for **a**, Bi2212 p=0.19; **b**, YBCO p=0.185; **c**, YBCO p=0.06. The green, red, orange, blue Gaussians and the region below the grey dashed line represent respectively the pure elastic, the CDF, the bond-stretching phonon modes, the phonon overtones and the paramagnons. In addition to them, used in the main text for the determination of the different CDF parameters, we have here also considered a Gaussian (in black) for the buckling phonons. At q_{CDF}, the intensity of this additional curve is much lower than the CDF Gaussian, and the resulting CDF parameters are effectively identical to those from the fit without buckling phonons (i.e., the variation of the CDF energy is below the uncertainty we have on this value).

SUPPLEMENTARY REFERENCES

[1] Arpaia, R. et al. Dynamical charge density fluctuations pervading the phase diagram of a Cu-based high-Tc superconductor. *Science* **365**, 906 (2019).

[2] Rossi, M. et al. Experimental determination of momentum-resolved electron-phonon coupling. *Phys. Rev. Lett.* **123**, 027001 (2019).

[3] Braicovich, L. et al. Determining the electron-phonon coupling in superconducting cuprates by resonant inelastic x-ray scattering: methods and results on $Nd_{1+x}Ba_{2-x}Cu_3O_{7-\delta}$. *Phys. Rev. Res.* **2**, 023231 (2021).

[4] Chaix, L. et al. Dispersive charge density wave excitations in $Bi_2Sr_2CaCu_2O_{8+\delta}$. *Nat. Phys.* **13**, 952–956 (2017).

[5] Lee, W. et al. Spectroscopic fingerprint of charge order melting driven by quantum fluctuations in a cuprate. *Nat. Phys.* **17**, 53–57 (2021).

[6] Huang, H.Y. et al. Quantum fluctuations of charge order induce phonon softening in a super-conducting cuprate. *Phys. Rev. X* **11**, 041038 (2021).

[7] Wang, Q. et al. Charge order lock-in by electron-phonon coupling in $La_{1.675}Eu_{0.2}Sr_{0.125}CuO_4$. *Sci. Adv.* **7**, eabg7394 (2021).
